# Lipedema prevalence and risk factors in Brazil

**DOI:** 10.1590/1677-5449.202101981

**Published:** 2022-05-23

**Authors:** Alexandre Campos Moraes Amato, Fernando Campos Moraes Amato, Juliana Lelis Spirandeli Amato, Daniel Augusto Benitti

**Affiliations:** 1 Amato, Instituto de Medicina Avançada, São Paulo, SP, Brasil.; 2 Valens Medical Center, Campinas, SP, Brasil.

**Keywords:** prevalence, questionnaires, lipedema, obesity, lymphedema

## Abstract

**Background:**

Lipedema is characterized as an abnormal deposition of fat in the buttocks and legs bilaterally that may be accompanied by swelling, pain, and tenderness. It is still often confused with more frequent conditions such as obesity and lymphedema. The estimated prevalence in Europe varies between 0.06% and 39%.

**Objectives:**

To evaluate the prevalence of lipedema and identify health factors related to it in the Brazilian population.

**Methods:**

Administration of a previously validated online screening questionnaire to a representative sample of the general population. The questionnaire was distributed and administered to anonymous volunteers representing the general Brazilian population using software designed for population analyses.

**Results:**

253 women answered the questionnaire, 12.3 ± 4% (Confidence Interval [CI] 95%) of whom presented symptoms compatible with a high probability of being diagnosed with lipedema. Furthermore, anxiety, depression, hypertension, and anemia were also correlated with a high probability of the diagnosis.

**Conclusions:**

The estimated prevalence of lipedema in the population of Brazilian women is 12.3%.

## INTRODUCTION

Lipedema was described for the first time in 1940 by doctors Edgar Van Nuys Allen, the cardiovascular surgeon known for the Allen test, and Edgar Alphonso Hines Jr. at the Mayo Clinic,^1^^,^^2^ in the Vascular Clinics sessions; which is why the condition is also known as Allen-Hines syndrome.^3^ Since then, lipedema has been defined as bilateral abnormal deposition of fat in the buttocks and legs, which may be accompanied by orthostatic edema.^1^^,^^2^ The pathophysiology and epidemiology of lipedema remain little understood.^4^^,^^5^ Moreover, lipedema was only recently included in the 11th revision of the International Classification of Diseases (ICD-11) (EF02.2 and BD93.1Y)^6^ and therefore is not yet part of the academic curriculum of medical degrees in Brazil, nor of the vascular specialty curriculum. It is thus still often confused with other more common conditions, such as obesity, gynoid lipodystrophy, and lymphedema,^7^^,^^8^ and is rarely diagnosed at the first medical consultation.^4^

**Figure 1:**
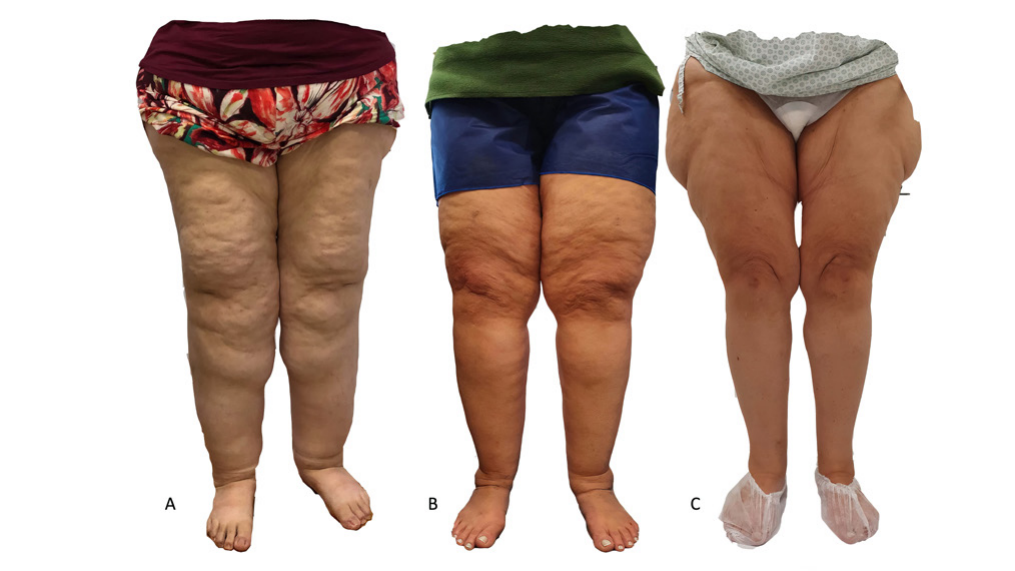
(A) Lipedema of buttocks and ankles; (B) Lipedema from buttocks to proximal leg; (C) Lipedema of pelvis, buttocks, and hips.

Diagnosis of lipedema is essentially clinical, defined as symmetrical disproportionate accumulation of fat in the lower limbs (Figure 1) accompanied by complaints of orthostatic edema^7^^,^^9^ and often by pain. It predominantly occurs in women.^5^ Imaging exams such as ultrasound,^10^ magnetic resonance,^11^ and computed tomography^12^ can confirm the diagnosis. Recently, Amato et al.^13^ published methodology for individual screening for lipedema using a self-administered questionnaire that showed excellent diagnostic accuracy,^14^ making it possible to estimate the prevalence of lipedema using Brazilian census data.^15^

The literature reports estimated prevalence rates of lipedema in the German population ranging from 0.06%^16^ to 39%.^7^^,^^17^^,^^18^ Since there has never been an evaluation of lipedema in the Brazilian population and considering the existence of Brazilian census projections for 2021,^19^ we conducted a Brazilian population study using the tools currently available.

The primary objective of this study was to assess the prevalence of lipedema in the Brazilian female population. A secondary objective was to identify risk factors such as symptoms or diseases associated with lipedema.

## METHODS

As previously proposed,^13^ the lipedema screening methodology used is based on the sum of points from a questionnaire administered in a population survey, indicating the probability of a diagnosis of lipedema in the study population.

The questionnaire was converted into an on-line digital version and questions were added to collect data on demographics and health indicators (weight, height, comorbidities, treatments undergone, symptoms, and surgery), using secure and appropriate software that has been validated^20^ for development and analysis of questionnaires (SurveyMonkey®, San Mateo, CA, United States). The questionnaire was administered to anonymous volunteers representative of the Brazilian general population.

### Patients

The sampling technique employed was randomization adjusted by representativeness of the population and was performed automatically by the specialized software used. The sample of the population selected to receive the questionnaire was distributed manually by selecting female sex and segmented by age, based on the proportions in the projections published in the 2021 census, with age groups weighted as follows: 20-29 years: 22%; 30-39 years: 24%; 40-49 years: 22%; 50-59 years: 18%; and 60-69 years: 14%. The participants included were women over the age of 18 years who were registered on the on-line survey platform. Males and women who did not digitally sign the consent form were excluded.

### Prediction model

The mathematical formula employed to calculate the probability of lipedema from total score has been published elsewhere.^13^ It employs a total score coefficient of 0.361 and a constant of -3.075: ${\left({\text{e}}^{-\left(\text{total score coefficient}+\text{constant}\right)}+1\right)}^{-1}$ .

### Definition of the diagnostic criterion

**Figure 2:**
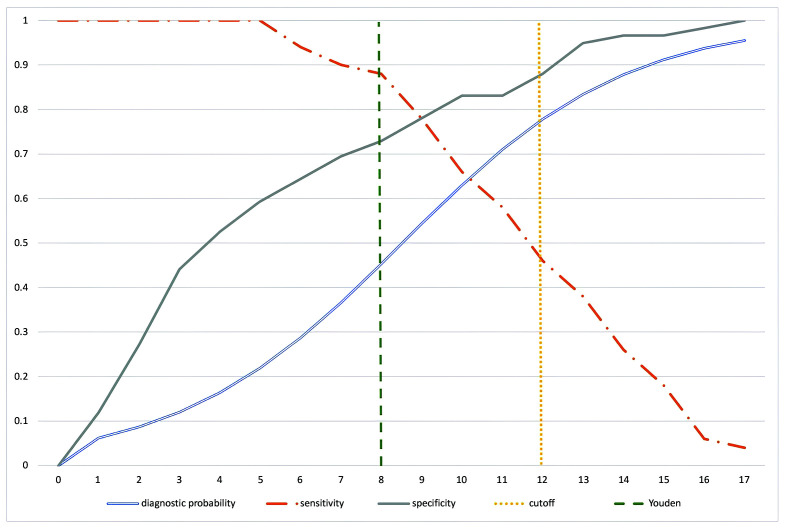
Study of sensitivity, specificity, and individual diagnostic probability against questionnaire scores. The vertical line indicates the cutoff selected for maximum specificity.

The total score cutoff method was used, with an area under the receiver operating curve (ROC curve) of 0.8615, which can be considered an excellent level of accuracy.^13^ Using the Youden index method (J = sensitivity + specificity - 1), a total score cutoff point of 8 would achieve sensitivity of 0.88 and specificity of 0.729, with a probability of lipedema diagnosis of 45.3% (95% confidence interval [95%CI]: 33.6%-57.6%) (Table 1). We took a more conservative approach, aiming to achieve specificity closer to 0.9, setting the cutoff at 12, at which point the probability of diagnosis of lipedema is 77.8% (95%CI: 64.2-87.3%) (Table 2 and Figure 2).

**Table 1:** Study to define the best cutoff point for the screening questionnaire.

**Cutoff point**	**Probability of diagnosis of lipedema**	**95% confidence interval**	**Sensitivity**	**Specificity**	**Prevalence in the study population**
5	21.9%	12.7-35.2%	1	0.593	51.0% (129)
6	28.7%	18.4-41.8%	0.94	0.644	42.3% (107)
7	36.6%	25.5-49.4%	0.9	0.695	37.2% (94)
8	45.3%	33.6-57.6%	0.88	0.729	30.4% (77)
9	54.3%	42.0-66.1%	0.78	0.780	24.9% (63)
10	63.0%	50.1-74.3%	0.66	0.831	19.4% (49)
11	71.0%	57.5-81.5%	0.58	0.831	15.0% (38)
**12**	**77.8%**	**64.2-87.3%**	**0.46**	**0.880**	**12.3% (31)**
13	83.4%	70.1-91.5%	0.38	0.949	7.9% (20)
14	87.8%	75.2-94.5%	0.26	0.966	4.7% (12)
15	91.2%	79.6-96.5%	0.18	0.966	4.7% (12)
16	93.7%	83.3-97.8%	0.06	0.983	3.2% (8)
17	95.5%	86.4-98.6%	0.04	1.000	2.4% (6)

**Table 2:** Statistical analysis of the diagnostic definition criterion.

	**Value**	**Lower limit**	**Upper limit**
**Sensitivity**	0.460	0.318	0.607
**Specificity**	0.881	0.771	0.951
**Positive predictive value**	0.767	0.598	0.856
**Negative predictive value**	0.658	0.513	0.834
**Positive likelihood ratio**	3.877	1.818	8.270
**Negative likelihood ratio**	0.613	0.467	0.805

### Statistical analysis

A sample size of 151 questionnaires was calculated to achieve a 95%CI, considering an 11% prevalence. After checking data consistency manually, descriptive statistics and frequencies were calculated using Excel (Microsoft), Wizard 2.0.5 (Evan Miller), and MedCalc. Cronbach’s Alpha was used to estimate the questionnaire’s reliability, the Kruskal-Wallis test was run and histograms were plotted for comparison of population subsets. A log-linear prediction model was applied to the screening questionnaire. Correlations between questionnaire variables were tested using Spearman coefficients and the Shapiro-Wilk test. Risk factors were compared between subsets using Pearson correlation coefficients, Spearman correlation coefficients, and the chi-square test (z scores). A statistical analysis of the prediction model employed has been described elsewhere.^13^ We adopted a statistical significance level of 0.05% for the correlations.

This study complies with the standards set out in National Health Council resolution 196/96 on research involving human beings and the Helsinki Declaration and was approved by the Plataforma Brasil Research Ethics Committee under protocol CAAE: 09590919.6.0000.0081, decision number 01032021.

## RESULTS

**Figure 3:**
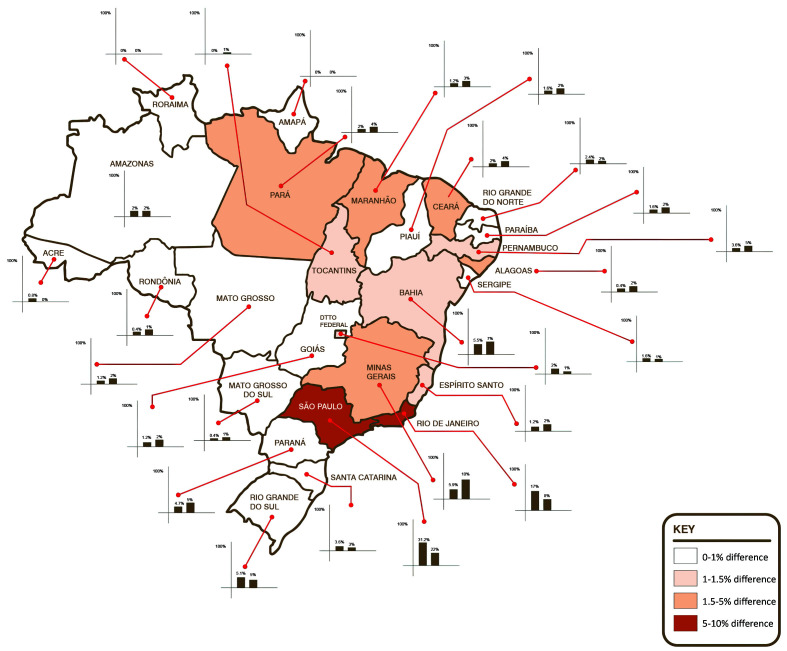
Geographical distribution of the sample studied compared to the population distribution, illustrating areas with greatest difference from the proportions of the 2021 census projection.

**Figure 4:**
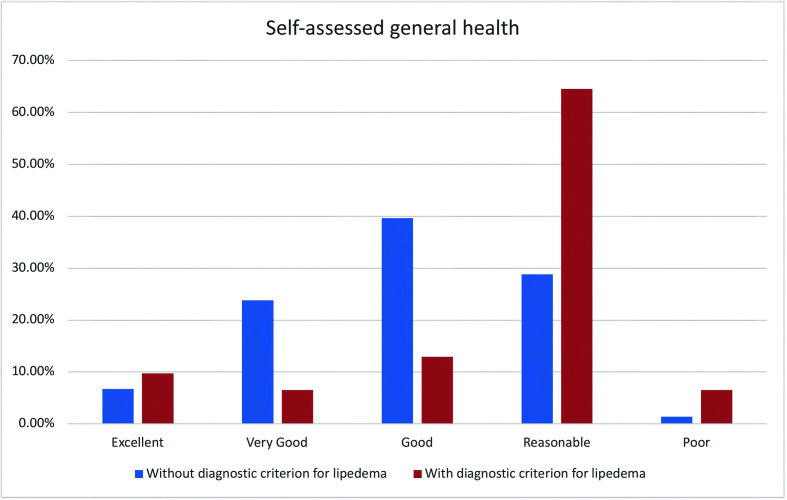
Comparison of self-assessed health of volunteers with and without lipedema diagnostic criterion.

The questionnaire was tested for reliability, achieving a Cronbach’s Alpha of 0.81 (95%CI: 0.7792). Two hundred and fifty-three women from all over Brazil answered the screening questionnaire, with a distribution representative of the Brazilian population (Kruskal-Wallis H 38.2642, N = 52, p < 0.00001) (Figure 3, Table 3), providing data on health-related factors and indices (Table 4, Table 5) and a general health self-assessment (Table 6). Mean body mass index (BMI) of the volunteers was 26.937 kg/cm ^2^ and the mean BMI of volunteers with scores positive for a diagnosis of lipedema was 27kg/cm.^2^ The mean age of the whole study population was 38.115 years (± standard deviation [SD] 12.4), while mean age of volunteers with the diagnostic criterion was 38.419 years (± SD 11.05); which are equivalent. The volunteers’ educational level was similar to the level predicted in the 2021census projection.^21^ There was a 5% dropout rate during completion of the questionnaire (n = 13) and the mean time taken to respond was 4 minutes and 18 seconds. It was observed that 12.3 ± 4% (95%CI, z score p < 0.001) of the study population met the criterion for diagnosis of lipedema. Table 5 lists the health-related factors studied. The subset of women with the lipedema diagnosis criterion reported the following conditions: arterial hypertension in 41.9% (positive correlation, z score p = 0.013), hypothyroidism in 19.4% (independent, z score p = 0.142), varicose veins and venous insufficiency in 35.5% (independent, z score p = 0.56), depression in 38.7% (positive correlation, z score p = 0.026), anxiety in 61.3% (positive correlation, z score p = 0.042), changes to bowel movements in 29% (independent, z score p = 0.068), knee disorders in 22.6% (independent, z score p = 0.182), hypercholesterolemia in 19.4% (independent, z score p = 0.952), lymphedema in 3.2% (independent, z score p = 0.103), anemia in 41.9% (positive correlation, z score p = 0.002), leg pain in 90.3% (positive correlation, z score p < 0.001), “water retention” in the legs in 64.5% (independent, z score p < 0.001), touch sensitivity in 35.5% (positive correlation, z score p < 0.001), swollen legs in 51.6% (positive correlation, z score p < 0.001), frequent leg bruising in 54.8% (positive correlation, z score p < 0.001), joint hypermobility in 9.7% (positive correlation, z score p < 0.001), knee pain in 58.1% (positive correlation, z score p = 0.007), feeling of heaviness in the legs in 51.6% (positive correlation, z score p < 0.001), burning sensations in the legs in 48.4% (positive correlation, z score p < 0.001), leg cramps in 35.5% (independent, z score p = 0.250), problems reducing leg weight or volume in 51.6% (positive correlation, z score p < 0.001), difficulty sleeping/poor sleep quality in 29% (independent, z score p = 0.392), prior varicose veins surgery in 48.4% (positive correlation, z score p < 0.001), bariatric surgery in 3.2% (independent, z score p = 0.983), and prior liposuction in 16.1% (positive correlation, z score p = 0.010). Table 6 lists the results for general self-assessed health status, which was rated as excellent health by 9.7% (independent, z score p = 0.553), very good health by 6.5% (negative correlation, z score p = 0.028), good health by 12.9% (negative correlation, z score p = 0.004), reasonable health by 64.5% (positive correlation, z score p < 0.001) and poor health by 6.5% (independent, z score p = 0.056) (Figure 4).

**Table 3:** Characteristics of the study population compared with the population projected by the 2021 census. Kruskal-Wallis (H 3.5735 N =16, p = 0.05871).

**Patient characteristics**	**Study population**	**2021 census projection**
Volunteers	253	212,854,215
Educational level^21^		
Secondary education, incomplete	2.4% (6)	6.7%
Secondary education, complete	29.2% (74)	25.1%
Primary education	4.0% (10)	8.0%
Technical college	7.5% (19)	-
Higher education, incomplete	11.5% (29)	4.8%
Higher education, complete	28.5% (72)	14.7%
Post-graduation	16.6% (42)	-

**Table 4:** Health-related factors associated with lipedema in volunteers with scores over the cutoff compared with all volunteers.

	**Volunteers with diagnostic criterion for lipedema**	**Volunteers without diagnostic criterion for lipedema**	**All volunteers**	**Correlation with lipedema diagnosis**
**Age**	38.419 years (± 11.05)	38.072 years (± 12.6)	38.115 years (± 12.4)	Kolmorov-Smirnov
				distribution equivalent, p = 0.696
				OR 0.951, SE 0.005
**BMI**	27.000 kg/cm^2^ (31)	26.920 kg/cm^2^ (222)	26.937 kg/cm^2^ (253)	Kolmorov-Smirnov
				distribution equivalent, p = 0.193
				OR 0.93, SE 0.007
**Underweight**	-	1.3% (3)	1.2% (3)	Independent (z score, p = 0.515)
				OR 1, SE 0
**Normal weight**	32.3% (10)	43.2% (96)	41.9% (106)	Independent (z score, p = 0.246)
				OR 0.104, SE 0.035
**Overweight**	41.9% (13)	32.4% (72)	33.6% (85)	Independent (z score, p = 0.294)
				0.181, SE 0.054
**Obesity I**	22.6% (7)	13.5% (30)	14.6% (37)	Independent (z score, p = 0.181)
				OR 0.233, SE 0.098
**Obesity II**	3.2% (1)	5.4% (12)	5.1% (13)	Independent (z score, p = 0.607)
				OR 0.083, SE 0.087
**Obesity III**	-	4.1% (9)	3.6% (9)	Independent (z score, p = 0.2540
				OR 0, SE 1,525E-9

BMI: body mass index; OR: odds ratios; SE: standard error.

**Table 5:** Self-report health factors.

	**Volunteers with diagnostic criterion for lipedema**	**Volunteers without diagnostic criterion for lipedema**	**All volunteers**	**Correlation with lipedema diagnosis**
**Arterial hypertension**	41.90% (13)	21.60% (48)	24.10% (61)	Not independent (z score, p = 0.013), positive correlation (Spearman)
				OR 0.271, SE 0.085
**Hypothyroidism**	19.40% (6)	10.36% (23)	11.50% (29)	Independent (z score, p = 0.141)
				OR 0.126, SE 0.027
**Varicose veins and venous insufficiency**	35.50% (11)	20.27% (45)	22.10% (56)	Independent (z score, p = 0.056)
				OR 0.244, SE 0.082
**Depression**	38.70% (12)	20.70% (46)	22.90% (58)	Not independent (z score, p = 0.026), positive correlation (Spearman)
				OR 0.108, SE 0.026
**Anxiety**	61.30% (19)	41.80% (93)	44.30% (112)	Not independent (z score, p = 0.042), positive correlation (Spearman)
				OR 0.093, SE 0.028
**Changed bowel movements**	29.00% (9)	15.70% (35)	17.40% (44)	Independent (z score, p = 0.068)
				OR 0.118, SE 0.027
**Knee problems/knee disease**	22.60% (7)	13.50% (30)	14.60% (37)	Independent (z score, p = 0.181)
				OR 0.233, SE 0.098
**Hypercholesterolemia**	19.40% (6)	19.80% (44)	19.80% (50)	Independent (z score, p = 0.951)
				OR 0.14, SE 0.03
**Lymphedema**	3.20% (1)	0.45% (1)	0.80% (2)	Independent (z score, p = 0.102)
				OR 0.136, SE 0.026
**Anemia**	41.90% (13)	17.50% (39)	20.60% (52)	Not independent (z score, p = 0.002), positive correlation (Spearman)
				OR 0.098, SE 0.0240
**Leg pain**	90.30% (28)	42.70% (95)	48.60% (123)	Not independent (z score, p < 0.001), positive correlation (Spearman)
				OR 0.295, SE 0.063

**“Water retention” in legs**	64.50% (20)	19.80% (44)	25.30% (64)	Not independent (z score, p < 0.001), positive correlation (Spearman)
				OR 0.455, SE 0.123
**Sensitivity to touch**	35.50% (11)	5.90% (13)	9.50% (24)	Not independent (z score, p < 0.001), positive correlation (Spearman)
				OR 0.846, SE 0.347
**Swollen legs**	51.60% (16)	7.60% (17)	13.00% (33)	Not independent (z score, p < 0.001), positive correlation (Spearman)
				OR 0.073, SE 0.0200
**Frequent bruising of the legs**	54.80% (17)	16.60% (37)	21.30% (54)	Not independent (z score, p < 0.001), positive correlation (Spearman)
				OR 0.459, SD 0.135
**Joint hypermobility**	9.70% (3)	0.45% (1)	1.60% (4)	Not independent (z score, p < 0.001), positive correlation (Spearman)
				OR 0.127, SD 0.025
**Knee pain**	58.10% (18)	33.30% (74)	36.40% (92)	Not independent (z score, p = 0.007), positive correlation (Spearman)
				OR 0.088, SD 0.025
**Feelings of heaviness in the legs**	51.60% (16)	22.90% (51)	26.50% (67)	Not independent (z score, p < 0.001, positive correlation (Spearman)
				OR 0.314, SD 0.090
**Burning sensations in the legs**	48.40% (15)	9.90% (22)	14.60% (37)	Not independent (z score, p < 0.001), positive correlation (Spearman)
				OR 0.682, SD 0.228
**Leg cramps**	35.50% (11)	25.60% (57)	26.90% (68)	Independent (z score, p = 0.249)
				OR 0.121, SD 0.029
**Difficulty losing weight or volume from legs**	51.60% (16)	17.60% (39)	21.70% (55)	Not independent (z score, p < 0.001), positive correlation (Spearman)
				OR 0.082, SD 0.022
**Difficulty sleeping/poor sleep**	29.00% (9)	36.90% (82)	36.00% (91)	Independent (z score, p = 0.390)
				OR 0.120, SD 0.039
**Prior varicose veins surgery**	48.40% (15)	12.10% (27)	16.60% (42)	Not independent (z score, p < 0.001), positive correlation (Spearman)
				OR 0.556, SD 0.179
**Bariatric surgery**	3.20% (1)	3.10% (7)	3.20% (8)	Independent (z score, p = 0.983)
				OR 0.143, SD 0.153
**Liposuction**	16.10% (5)	4.50% (10)	5.90% (15)	Not independent (z score, p = 0.034), positive correlation (Spearman)
				OR 0.123, SD 0.025

OR: odds ratios; SE: standard error; SD: standard deviation.

**Table 6:** Self-reported general health status.

	**Volunteers with diagnostic criterion for lipedema**	**Volunteers without diagnostic criterion for lipedema**	**All volunteers**	**Correlation with diagnosis of lipedema**
**Excellent**	9.7% (3)	6.7% (15)	7.1% (18)	Independent (z score, p = 0.553)
				OR 0.200, SD 0.126
**Very good**	6.5% (2)	23.8% (53)	21.7% (55)	Not independent (z score, p = 0.028), negative correlation (Spearman)
				OR 0.038, SD 0.027
**Good**	12.9% (4)	39.6% (88)	36.4% (92)	Not independent (z score, p = 0.004), negative correlation (Spearman)
				OR 1, SD 0
**Regular**	64.5% (20)	28.8% (63)	32.8% (83)	Not independent (z score, p < 0.001), positive correlation (Spearman)
				OR 0.317, SD 0.081
**Poor**	6.5% (2)	1.4% (3)	2.0% (5)	Independent (z score, p = 0.056)
				OR 0.667, SD 0.609

OR: odds ratios; SD: standard deviation.

## DISCUSSION

The automated methodology used to select a sample of the total population, performed by the SurveyMonkey^®^ software (California, United States), has previously proven effective and achieved results within a 10% margin of error^20^ in other populations, as was the case with the distribution obtained (Figure 3). The questionnaire demonstrated appropriate reliability.^22^ Similarity between the population sample studied and the general population was demonstrated (Table 3). There was a minor difference (p = 0.05871; Kruskal-Wallis) between the study population and the census projection in terms of educational level, possibly because of selection of respondents who have access to the internet, inherent to the methodology proposed. The time taken to complete the questionnaire was 18% slower than for the original questionnaire^13^ (3 minutes and 38 seconds), which can be explained by the additional questions on health-related factors and indices, while the dropout rate was low.

The most widely-accepted lipedema prevalence reported in the literature was estimated by Földi and Földi, who extrapolated their clinical experience to the general population,^23^ estimating that up to 11% of the female population may have lipedema. In another study, Fife et al.^7^ found 10 to 20%.^7^ According to Schwahn-Schreiber and Marshall,^18^ the prevalence in Germany of all stages of lipedema is as great as 39% of the population. Other reports of the percentage of patients seen at lymphedema clinics who have lipedema vary from 8 to 18.8%.^16^^,^^24^^,^^25^ However, none of the studies mentioned reporting lipedema prevalence employed a validated tool. When diagnosed, many patients remember other relatives who have characteristics of the disease, with family incidence varying from 16 to 45% in the literature.^7^^,^^26^

Considering the methodology proposed, simply increasing the cutoff point (a right-shift on the graph, Figure 2) for the questionnaire score increases the individual probability of lipedema diagnosis, but the lower sensitivity that results could increase the number of false negatives, thereby reducing the number of patients identified in the population. An equilibrium between specificity and sensitivity is very important when estimating prevalence. The Youden index^27^ is the most frequently suggested method, but in the present study it was inadequate for determination of the best cutoff point. Since this is a population study, we decided to increase the specificity of the test and the individual probability of diagnosis, so that the prevalence measured was more conservative. We achieved a diagnostic probability of 77.8% (95%CI: 64.2-87.3%) for the tool employed, with specificity of 0.88 (95%CI: 0.77-0.95) and sensitivity of 0.46 (95%CI: 0.31-0.6) (Table 2 and Figure 2). With these parameters, the prevalence in the female Brazilian population was 12.3% (Table 1). The 2021 census projection estimates that there are around 100.5 million women in Brazil, 71,739,082 aged from 18 to 69 years. Applying the estimated prevalence, we estimate that there are around 8.8 million women aged 18 to 69 years who have symptoms suggestive of lipedema.

Dudek et al.^6^ used a similar questionnaire to investigate a group of Polish women with suspected lipedema, estimating mean BMI at 30.8 kg/cm^2^ (SD = 7.1), with 76.5% classified as overweight (26.5%) or obese (50%). Our study observed that 67.5% of the women with lipedema had BMI greater than 25 kg/cm^2^, with a mean BMI of 27kg/cm^2^. Elevated BMI makes diagnosis more difficult because of the complexity of differentiation from common obesity. It can be difficult to distinguish between lipedema and other variations of anatomic fat deposition,^28^ since the disproportionate fat distribution typical of lipedema can easily be confused with gynecoid disproportion or pear-shaped obesity,^29^ which do not have the same symptomology as lipedema. The Polish study also identified hypothyroidism in 31.6%,^30^ while another study, with a Dutch population, reported 11.7%.^31^ Among the Brazilian women, we identified this disorder in 19.4%, with no correlation between volunteers with or without symptoms suggestive of lipedema.

The self-reported lymphedema rate was 30.6% in the Dutch sample but was only mentioned by 3.2% of the Brazilian women. Venous insufficiency was present in 20.4% of the Polish sample and in 35.5% of the Brazilian women, among whom it did not correlate with groups, although 48.4% reported prior varicose veins surgery, which was positively correlated. Arthritis was identified in 20.4% of the Polish sample. Knee pain was self-reported in 58.1% of the Brazilian women with lipedema. Arterial hypertension was identified in 4%^6^ of the Polish lipedema patients and 18.4%^31^ of the Dutch sample, whereas it was mentioned by 41.9% of the Brazilians, with a positive correlation. Although joint hypermobility characteristic of Ehlers-Danlos Syndrome^32^ has been associated with lipedema in previous publications,^33^^,^^34^ it was not common in the Polish^6^ or Dutch populations;^31^ while among the Brazilian women, the frequency was low, but was positively correlated.

With relation to associated symptoms reported by the participants, putting weight on in the arms and legs easily was mentioned by 99% of the Polish sample, feelings of heavy legs by 96.9%, frequent ecchymosis by 90.8%, and difficulty losing weight from arms and legs by 86.8%. Difficulty losing limb weight and volume was endorsed by 51.6% of the Brazilian women, with a positive correlation. We observed that feelings of heaviness were endorsed by 51.6%, while the 54.8% rate of frequent ecchymosis exhibited a positive correlation, as did burning sensations in the legs. Sensitivity to touch was mentioned on the questionnaire by 35.5% of the Brazilian women, leg pains by 90.3%, swelling by 51.6%, and feelings of water retention in the legs by 64.5%, which are characteristics that fit the current diagnostic criteria.^35^

In the literature,^6^^,^^31^^,^^36^ 42 to 59.2% report depressive symptoms and anxiety. We found self-reported depression in 38.7% of the Brazilian women with lipedema and anxiety in 61.3%, both with positive correlations. Anemia, which has not previously been reported in other similar studies, was mentioned by 41.9% of these volunteers and was positively correlated with the disease.

In the Polish analysis,^6^ 34.7% of patients reported good or very good quality of life, while 20.4% reported poor or very poor quality of life. Along the same lines, we found a significant increase in Brazilian women with symptoms suggestive of lipedema who reported regular or poor health, with a positive correlation, clearly illustrating the extent to which lipedema-related symptoms have a negative effect on perceived general health (Figure 4).

Increased coverage of lipedema in the media can result in a bias towards lipedema diagnosis, and it is necessary to consider differential diagnostic possibilities,^37^ such as lipohypertrophy, obesity, lymphedema, phlebolymphedema, stasis edema, and fibromyalgia.^29^ Notwithstanding, lipedema is still very much underdiagnosed.^38^ Lipedema symptoms and complaints can be considered subjective, particularly during the early phases, and may be confounded with other diseases that are seen more often in vascular surgery clinics, to the extent that it is necessary to standardize the assessment criteria to increase the objectivity of diagnosis of this condition. It is therefore important to develop and validate instruments capable of assessing the clinical impact of lipedema and of supporting definitive diagnosis. If patients with lipedema are not correctly diagnosed, treatment of the disease is delayed, allowing it to progress.^38^ The following symptoms and elements of medical history associated with lipedema were positively correlated: leg pain, “water retention”, sensitivity to touch, swollen legs, frequent bruising of the legs, joint hypermobility, knee pain, feelings of heaviness in the legs, burning sensations in the legs, difficulty losing weight or volume from the legs, prior varicose veins surgery, and liposuction.

This is the first study to survey the prevalence and characteristics of lipedema in the Brazilian population and was designed to obtain the prevalence of lipedema in Brazil. It has some limitations, including the cyclical nature of lipedema symptoms^5^ which can lead to underestimation of the prevalence in the population. Additionally, the study was conducted on-line, using self-assessment – which limits socioeconomic status – and without medical supervision or diagnostic confirmation.

## CONCLUSIONS

In this study, we observed that the prevalence of lipedema in the population of Brazilian women is 12.3%. We estimate, conservatively, that 8.8 million adult Brazilian women aged 18 to 69 years may have symptoms suggestive of a diagnosis of lipedema. Anxiety, depression, arterial hypertension, and anemia appear to be associated with lipedema.
