# Supplementary material for: Lipedema prevalence and risk factors in Brazil
Source: J Vasc Bras. 2022 May 23;21:e20210198. doi: 10.1590/1677-5449.202101981 (PMC9136687; doi:10.1590/1677-5449.202101981)
Supplement: Translation

# Translation: Prevalência e fatores de risco para lipedema no Brasil

**DOI:** 10.1590/1677-5449.202101982

**Authors:** Alexandre Campos Moraes Amato, Fernando Campos Moraes Amato, Juliana Lelis Spirandeli Amato, Daniel Augusto Benitti

**Affiliations:** 1 Amato, Instituto de Medicina Avançada, São Paulo, SP, Brasil.; 2 Valens Medical Center, Campinas, SP, Brasil.

**Keywords:** prevalência, questionários, lipedema, obesidade, linfedema

## Abstract

**Contexto:**

O lipedema é caracterizado por deposição anormal de gordura em glúteos e pernas bilateralmente, que pode ser acompanhada por edema, dor e sensibilidade ao toque. Ainda é frequentemente confundido com condições mais frequentes, como obesidade e linfedema. A prevalência estimada na Europa varia entre 0,06% e 39%.

**Objetivos:**

Avaliar a prevalência do lipedema na população brasileira e identificar fatores de saúde relacionados a essa doença.

**Métodos:**

Foi aplicado um questionário de rastreamento on-line, previamente validado em amostra representativa da população geral. O questionário de rastreamento foi distribuído e aplicado em voluntárias anônimas representativas da população geral brasileira por *software* dedicado a análises populacionais.

**Resultados:**

Um total de 253 mulheres respondeu ao questionário, e 12,3 ± 4% (intervalo de confiança de 95%) apresentaram sintomatologia compatível com alta probabilidade de diagnóstico de lipedema. Ansiedade, depressão, hipertensão e anemia foram correlacionadas com a alta probabilidade diagnóstica da doença.

**Conclusões:**

A prevalência estimada do lipedema na população de mulheres brasileiras é de 12,3%.

## INTRODUÇÃO

O lipedema foi descrito pela primeira vez em 1940 pelos doutores Edgar Van Nuys Allen, cirurgião cardiovascular conhecido pelo teste de Allen, e Edgar Alphonso Hines Jr. na Mayo Clinic^1^^,^^2^, na sessão *Vascular Clinics*; por isso, a síndrome é denominada Allen-Hines^3^. Desde então, o lipedema foi caracterizado como uma deposição anormal de gordura em glúteos e pernas bilateralmente, que pode ser acompanhada por edema ortostático^1^^,^^2^. A fisiopatologia e a epidemiologia do lipedema ainda são pouco compreendidas^4^^,^^5^. Além disso, o lipedema só foi incluído recentemente na 11ª revisão da Classificação Internacional de Doenças (CID-11) (EF02.2 e BD93.1Y)^6^, de forma que ainda não faz parte do currículo médico acadêmico brasileiro nem do currículo vascular especializado. Sendo assim, é ainda frequentemente confundido com outras condições mais frequentes, como a obesidade, lipodistrofia ginoide e linfedema^7^^,^^8^, e raramente é diagnosticado na primeira consulta médica^4^.

O diagnóstico de lipedema é essencialmente clínico, sendo definido pela desproporção simétrica do acúmulo de gordura nas extremidades inferiores (Figura 1) acompanhada por queixas de edema ortostático^7^^,^^9^ e, frequentemente, por dor. Ocorre predominantemente em mulheres^5^. Apesar disso, exames de imagem como ultrassom^10^, ressonância magnética^11^ e tomografia computadorizada^12^ podem corroborar o diagnóstico. Recentemente, Amato et al.^13^ publicaram metodologia para rastreamento individual do lipedema com questionário de autoaplicação, que mostrou excelente acurácia diagnóstica^14^, abrindo a possibilidade de estimar a prevalência do lipedema com os dados do censo brasileiro^15^.

**Figura 1:** (A) Lipedema em região de glúteos ao tornozelo; (B) Lipedema em região de glúteos até região proximal de perna; (C) Lipedema em região de pélvis, glúteos e quadris.

A literatura relata estimativas da prevalência de lipedema na população alemã entre 0,06%^16^ e 39%^7^^,^^17^^,^^18^. Tendo em vista que não existe nenhuma avaliação populacional sobre lipedema no Brasil e considerando a existência de projeção do censo brasileiro para 2021^19^, realizamos o estudo populacional brasileiro com as ferramentas atualmente disponíveis.

O estudo teve como objetivo primário a avaliação da prevalência do lipedema na população feminina brasileira. Secundariamente, pretendeu identificar fatores de risco como sintomas ou doenças associadas ao lipedema.

## MÉTODOS

Foi utilizada a metodologia previamente proposta por somatória de pontos do questionário de rastreamento de lipedema anteriormente publicado^13^ para caracterização da probabilidade do diagnóstico do lipedema na população estudada, aplicado em inquérito populacional.

O questionário foi convertido em versão digital on-line, e foram acrescentados perguntas demográficas e fatores e índices de saúde (peso, altura, comorbidades, tratamentos realizados, sintomas e cirurgia), com *software* seguro, apropriado e validado^20^ para desenvolvimento e análise de questionários (SurveyMonkey®, San Mateo, CA, EUA). Foi aplicado em voluntárias anônimas representativas da população geral brasileira.

### Casuística

A técnica de amostragem por randomização ajustada para representatividade da população foi realizada de forma automatizada por *software* especializado. A segmentação da amostra da população a receber o questionário foi distribuída manualmente por seleção de gênero feminino e segmentação da idade, fundamentada na proporção da projeção publicada pelo censo de 2021, assim sendo a ponderação de idade considerada: 20-29 anos: 22%; 30-39 anos: 24%; 40-49 anos: 22%; 50-59 anos: 18%; e 60-69 anos: 14%. Foram incluídas mulheres com idade maior que 18 anos e membros da plataforma de pesquisa on-line. Foram excluídos indivíduos do sexo masculino e mulheres que não assinaram digitalmente o termo de consentimento.

### Modelo de predição

A fórmula matemática utilizada para calcular a probabilidade do lipedema com os pontos totais foi previamente publicada^13^ e emprega o coeficiente dos pontos totais 0,361 e a constante de -3,075: ${\left({\text{e}}^{-\left(\text{coef pontos totais}+\text{constante}\right)}+1\right)}^{-1}$

### Definição do critério diagnóstico

Optou-se pela avaliação da pontuação, que possui curva característica de operação de receptor (*ROC curve*) com área abaixo da curva de 0,8615, considerada de excelente acurácia^13^. Segundo o método do índice de Youden (J = sensibilidade + especificidade - 1), o ponto de corte na somatória de pontos seria 8, com sensibilidade de 0,88 e especificidade de 0,729, e a probabilidade de diagnóstico de lipedema é de 45,3% (intervalo de confiança de 95% [IC95%]: 33,6%-57,6%) (Tabela 1). Optamos, de forma conservadora, por atingir especificidade mais próxima de 0,9, com nota de corte de 12, sendo que, nesse caso, a probabilidade de diagnóstico de lipedema é de 77,8% (IC95%: 64,2-87,3%) (Tabela 2, Figura 2).

**Tabela 1:** Estudo do melhor ponto de corte para o questionário de rastreamento.

**Ponto de corte na pontuação**	**Probabilidade do diagnóstico de lipedema**	**Intervalo de confiança de 95%**	**Sensibilidade**	**Especificidade**	**Prevalência na população estudada**
5	21,9%	12,7-35,2%	1	0,593	51,0% (129)
6	28,7%	18,4-41,8%	0,94	0,644	42,3% (107)
7	36,6%	25,5-49,4%	0,9	0,695	37,2% (94)
8	45,3%	33,6-57,6%	0,88	0,729	30,4% (77)
9	54,3%	42,0-66,1%	0,78	0,780	24,9% (63)
10	63,0%	50,1-74,3%	0,66	0,831	19,4% (49)
11	71,0%	57,5-81,5%	0,58	0,831	15,0% (38)
**12**	**77,8%**	**64,2-87,3%**	**0,46**	**0,880**	**12,3% (31)**
13	83,4%	70,1-91,5%	0,38	0,949	7,9% (20)
14	87,8%	75,2-94,5%	0,26	0,966	4,7% (12)
15	91,2%	79,6-96,5%	0,18	0,966	4,7% (12)
16	93,7%	83,3-97,8%	0,06	0,983	3,2% (8)
17	95,5%	86,4-98,6%	0,04	1,000	2,4% (6)

**Tabela 2:** Avaliação estatística do critério de definição diagnóstica.

	**Valor**	**Limite inferior**	**Limite superior**
**Sensibilidade**	0,460	0,318	0,607
**Especificidade**	0,881	0,771	0,951
**Valor preditivo positivo**	0,767	0,598	0,856
**Valor preditivo negativo**	0,658	0,513	0,834
**Razão de verossimilhança positiva**	3,877	1,818	8,270
**Razão de verossimilhança negativa**	0,613	0,467	0,805

**Figura 2:** Estudo da sensibilidade, especificidade e probabilidade diagnóstica individual segundo pontuação no questionário. A linha vertical representa pontuação de corte selecionada para maior especificidade.

### Análise estatística

Foi calculado o tamanho amostral de 151 questionários para IC95%, considerando prevalência de 11%. Após verificação manual da consistência dos dados, foi realizada análise estatística descritiva e de frequências com os *softwares* Excel (Microsoft), Wizard 2.0.5 (Evan Miller) e MedCalc. O alfa de Cronbach foi analisado para estimar a confiabilidade do questionário, e Kruskal-Wallis e histogramas foram empregados para comparação populacional. O modelo de predição aplicado no questionário de rastreamento foi log-linear. A correlação entre variáveis do questionário foi avaliada por correlação de Spearman e teste de Shapiro-Wilk. A comparação dos fatores de risco entre população foi avaliada pelo coeficiente de correlação de Pearson, Spearman e qui-quadrado (*z-score*). A análise estatística do modelo de predição empregada foi descrita previamente^13^. Assumimos, para as correlações, um nível de significância estatística de 0,05%.

O presente estudo segue as normas do Conselho Nacional de Saúde, referentes à resolução n.˚ 196/96 sobre pesquisa envolvendo seres humanos; também segue a declaração de Helsinki e foi aprovado pelo Comitê de Ética em Pesquisa da Plataforma Brasil sob protocolo CAAE: 09590919.6.0000.0081 e parecer 01032021.

## RESULTADOS

O questionário foi avaliado em confiabilidade e apresentou alfa de Cronbach 0,81 (IC95%: 0,7792). Duzentos e cinquenta e três mulheres de todo Brasil responderam ao questionário de rastreamento, com distribuição representativa da população brasileira (Kruskal-Wallis H 38,2642, N = 52, p < 0,00001) (Figura 3, Tabela 3), preenchendo os fatores e índices relacionados à saúde (Tabela 4, Tabela 5) e avaliação geral de saúde (Tabela 6). O índice de massa corpórea (IMC) médio de todas as voluntárias foi de 26,937 kg/cm^2^ e das voluntárias com pontuação positiva para o diagnóstico foi de 27kg/cm^2^. A idade média da população estudada foi de 38,115 anos (± desvio padrão [DP] 12,4) para todas as voluntárias, enquanto para as voluntárias com critério diagnóstico foi de 38,419 anos (± DP 11,05); portanto, equivalentes entre si. O nível de escolaridade entre as voluntárias foi semelhante ao identificado na projeção do censo de 2021^21^. O preenchimento do questionário apresentou taxa de desistência de 5% (13) e tempo médio para completá-lo de 4 minutos e 18 segundos. Identificou-se 12,3 ± 4% (IC95%, z-score p < 0,001) da população estudada com critérios para o diagnóstico de lipedema. Os fatores de saúde estudados estão descritos na Tabela 5, sendo que, no grupo de mulheres com critério diagnóstico de lipedema, foram relatadas as seguintes condições: hipertensão arterial em 41,9% (correlação positiva, *z-score* p = 0,013), hipotireoidismo em 19,4% (independente, *z-score* p = 0,142), varizes e insuficiência venosa em 35,5% (independente, *z-score* p = 0,56), depressão em 38,7% (correlação positiva, *z-score* p = 0,026), ansiedade em 61,3% (correlação positiva, *z-score* p = 0,042), alteração no hábito intestinal em 29% (independente, *z-score* p = 0,068), alterações geniculares em 22,6% (independente, *z-score* p = 0,182), hipercolesterolemia em 19,4% (independente, *z-score* p = 0,952), linfedema em 3,2% (independente, *z-score* p = 0,103), anemia em 41,9% (correlação positiva, *z-score* p = 0,002), dor nas pernas em 90,3% (correlação positiva, *z-score* p < 0,001), “retenção de líquido” nas pernas em 64,5% (independente, *z-score* p < 0,001), sensibilidade ao toque em 35,5% (correlação positiva, *z-score* p < 0,001), inchaço/edema nas pernas em 51,6% (correlação positiva, *z-score* p < 0,001), roxos frequentes nas pernas em 54,8% (correlação positiva, *z-score* p < 0,001), hipermobilidade das articulações em 9,7% (correlação positiva, *z-score* p < 0,001), dor no joelho em 58,1% (correlação positiva, *z-score* p = 0,007), sensação de peso nas pernas em 51,6% (correlação positiva, *z-score* p < 0,001), queimação nas pernas em 48,4% (correlação positiva, *z-score* p < 0,001), câimbras nas pernas em 35,5% (independente, *z-score* p = 0,250), dificuldade para perder peso ou volume nas pernas em 51,6% (correlação positiva, *z-score* p < 0,001), dificuldade no sono/sono ruim em 29% (independente, *z-score* p = 0,392), cirurgia prévia de varizes em 48,4% (correlação positiva, *z-score* p < 0,001), cirurgia bariátrica em 3,2% (independente, *z-score* p = 0,983) e lipoaspiração prévia em 16,1% (correlação positiva, *z-score* p = 0,010). A autoavaliação geral de saúde está descrita na Tabela 6, sendo relatadas excelente saúde em 9,7% (independente, *z-score* p = 0,553), muito boa saúde em 6,5% (correlação negativa, *z-score* p = 0,028), boa saúde em 12,9% (correlação negativa, *z-score* p = 0,004), saúde razoável em 64,5% (correlação positiva, *z-score* p < 0,001) e saúde ruim em 6,5% (independente, *z-score* p = 0,056) (Figura 4).

**Figura 3:** Distribuição geográfica da amostra estudada comparada à distribuição populacional, evidenciando áreas com maior desvio da proporção da projeção do censo de 2021.

**Tabela 3:** Características da população estudada comparada com a população projetada pelo censo de 2021. Kruskal-Wallis (H 3,5735 N =16, p = 0,05871).

**Característica da paciente**	**População estudada**	**Projeção do censo de 2021**
Voluntárias	253	212.854.215
Nível de escolaridade^21^		
Ensino médio incompleto	2,4% (6)	6,7%
Ensino médio completo	29,2% (74)	25,1%
Ensino fundamental	4,0% (10)	8,0%
Ensino técnico	7,5% (19)	-
Ensino superior incompleto	11,5% (29)	4,8%
Ensino superior completo	28,5% (72)	14,7%
Pós-graduação	16,6% (42)	-

**Tabela 4:** Fatores de saúde associados ao lipedema nas voluntárias acima da pontuação de corte comparada com todas as voluntárias.

	**Voluntárias com critério diagnóstico para lipedema**	**Voluntárias sem critério diagnóstico para lipedema**	**Total de voluntárias**	**Correlação com diagnóstico do lipedema**
**Idade**	38,419 anos (± 11,05)	38,072 anos (± 12,6)	38,115 anos (± 12,4)	Distribuição equivalente
				Kolmorov-Smirnov, p = 0,696
				OR 0,951, EP 0,005
**IMC**	27,000 kg/cm^2^ (31)	26,920 kg/cm^2^ (222)	26,937 kg/cm^2^ (253)	Distribuição equivalente
				Kolmorov-Smirnov, p = 0,193
				OR 0,93, EP 0,007
**Abaixo do peso**	-	1,3% (3)	1,2% (3)	Independente (*z-score*, p = 0,515)
				OR 1, EP 0
**Peso normal**	32,3% (10)	43,2% (96)	41,9% (106)	Independente (*z-score*, p = 0,246)
				OR 0,104, EP 0,035
**Sobrepeso**	41,9% (13)	32,4% (72)	33,6% (85)	Independente (*z-score*, p = 0,294)
				0,181, EP 0,054
**Obesidade I**	22,6% (7)	13,5% (30)	14,6% (37)	Independente (*z-score*, p = 0,181)
				OR 0,233, EP 0,098
**Obesidade II**	3,2% (1)	5,4% (12)	5,1% (13)	Independente (*z-score*, p = 0,607)
				OR 0,083, EP 0,087
**Obesidade III**	-	4,1% (9)	3,6% (9)	Independente (*z-score*, p = 0,2540
				OR 0, EP 1,525E-9

IMC: índice de massa corpórea; OR: razão de chances; EP: erro padrão.

**Tabela 5:** Fatores de saúde autorrelatados.

	**Voluntárias com critério diagnóstico para lipedema**	**Voluntárias sem critério diagnóstico para lipedema**	**Total de voluntárias**	**Correlação com diagnóstico do lipedema**
**Hipertensão arterial**	41,90% (13)	21,60% (48)	24,10% (61)	Não independente (*z-score*, p = 0,013), correlação positiva (Spearman)
				OR 0,271, EP 0,085
**Hipotireoidismo**	19,40% (6)	10,36% (23)	11,50% (29)	Independente (*z-score*, p = 0,141)
				OR 0,126, EP 0,027
**Varizes e insuficiência venosa**	35,50% (11)	20,27% (45)	22,10% (56)	Independente (*z-score*, p = 0,056)
				OR 0,244, EP 0,082
**Depressão**	38,70% (12)	20,70% (46)	22,90% (58)	Não independente (*z-score*, p = 0,026), correlação positiva (Spearman)
				OR 0,108, EP 0,026
**Ansiedade**	61,30% (19)	41,80% (93)	44,30% (112)	Não independente (*z-score*, p = 0,042), correlação positiva (Spearman)
				OR 0,093, EP 0,028
**Alteração do hábito intestinal**	29,00% (9)	15,70% (35)	17,40% (44)	Independente (*z-score*, p = 0,068)
				OR 0,118, EP 0,027
**Alterações geniculares/doença nos joelhos**	22,60% (7)	13,50% (30)	14,60% (37)	Independente (*z-score*, p = 0,181)
				OR 0,233, EP 0,098
**Hipercolesterolemia**	19,40% (6)	19,80% (44)	19,80% (50)	Independente (*z-score*, p = 0,951)
				OR 0,14, EP 0,03
**Linfedema**	3,20% (1)	0,45% (1)	0,80% (2)	Independente (*z-score*, p = 0,102)
				OR 0,136, EP 0,026
**Anemia**	41,90% (13)	17,50% (39)	20,60% (52)	Não independente (*z-score*, p = 0,002), correlação positiva (Spearman)
				OR 0,098, EP 0,0240
**Dor nas pernas**	90,30% (28)	42,70% (95)	48,60% (123)	Não independente (*z-score*, p < 0,001), correlação positiva (Spearman)
				OR 0,295, EP 0,063

**“Retenção de líquido” nas pernas**	64,50% (20)	19,80% (44)	25,30% (64)	Não independente (*z-score*, p < 0,001), correlação positiva (Spearman)
				OR 0,455, EP 0,123
**Sensibilidade ao toque**	35,50% (11)	5,90% (13)	9,50% (24)	Não independente (*z-score*, p < 0,001), correlação positiva (Spearman)
				OR 0,846, EP 0,347
**Inchaço/edema nas pernas**	51,60% (16)	7,60% (17)	13,00% (33)	Não independente (*z-score*, p < 0,001), correlação positiva (Spearman)
				OR 0,073, EP 0,0200
**Roxos frequentes nas pernas**	54,80% (17)	16,60% (37)	21,30% (54)	Não independente (*z-score*, p < 0,001), correlação positiva (Spearman)
				OR 0,459, DP 0,135
**Hipermobilidade das articulações**	9,70% (3)	0,45% (1)	1,60% (4)	Não independente (*z-score*, p < 0,001), correlação positiva (Spearman)
				OR 0,127, DP 0,025
**Dor no joelho**	58,10% (18)	33,30% (74)	36,40% (92)	Não independente (*z-score*, p = 0,007), correlação positiva (Spearman)
				OR 0,088, DP 0,025
**Sensação de peso nas pernas**	51,60% (16)	22,90% (51)	26,50% (67)	Não independente (*z-score*, p < 0,001, correlação positiva (Spearman)
				OR 0,314, DP 0,090
**Queimação nas pernas**	48,40% (15)	9,90% (22)	14,60% (37)	Não independente (*z-score*, p < 0,001), correlação positiva (Spearman)
				OR 0,682, DP 0,228
**Câimbras nas pernas**	35,50% (11)	25,60% (57)	26,90% (68)	Independente (*z-score*, p = 0,249)
				OR 0,121, DP 0,029
**Dificuldade para perder peso ou volume nas pernas**	51,60% (16)	17,60% (39)	21,70% (55)	Não independente (*z-score*, p < 0,001), correlação positiva (Spearman)
				OR 0,082, DP 0,022
**Dificuldade no sono/sono ruim**	29,00% (9)	36,90% (82)	36,00% (91)	Independente (*z-score*, p = 0,390)
				OR 0,120, DP 0,039
**Cirurgia prévia de varizes**	48,40% (15)	12,10% (27)	16,60% (42)	Não independente (*z-score*, p < 0,001), correlação positiva (Spearman)
				OR 0,556, DP 0,179
**Cirurgia bariátrica**	3,20% (1)	3,10% (7)	3,20% (8)	Independente (*z-score*, p = 0,983)
				OR 0,143, DP 0,153
**Lipoaspiração**	16,10% (5)	4,50% (10)	5,90% (15)	Não independente (*z-score*, p = 0,034), correlação positiva (Spearman)
				OR 0,123, DP 0,025

OR: razão de chances; EP: erro padrão; DP: desvio padrão.

**Tabela 6:** Avaliação geral autorreportada da saúde.

	**Voluntárias com critério diagnóstico para lipedema**	**Voluntárias sem critério diagnóstico para lipedema**	**Total de voluntárias**	**Correlação com diagnóstico do lipedema**
**Excelente**	9,7% (3)	6,7% (15)	7,1% (18)	Independente (*z-score*, p = 0,553)
				OR 0,200, DP 0,126
**Muito boa**	6,5% (2)	23,8% (53)	21,7% (55)	Não independente (*z-score*, p = 0,028), correlação negativa (Spearman)
				OR 0,038, DP 0,027
**Boa**	12,9% (4)	39,6% (88)	36,4% (92)	Não independente (*z-score*, p = 0,004), correlação negativa (Spearman)
				OR 1, DP 0
**Razoável**	64,5% (20)	28,8% (63)	32,8% (83)	Não independente (*z-score*, p < 0,001), correlação positiva (Spearman)
				OR 0,317, DP 0,081
**Ruim**	6,5% (2)	1,4% (3)	2,0% (5)	Independente (*z-score*, p = 0,056)
				OR 0,667, DP 0,609

OR: razão de chances; DP: desvio padrão.

**Figura 4:** Comparação da autoavaliação de saúde entre voluntárias com e sem critério diagnóstico de lipedema.

## DISCUSSÃO

A metodologia automatizada aplicada para seleção de amostra da população total, pelo *software* SurveyMonkey^®^ (Califórnia, EUA), mostrou-se previamente eficiente e abaixo da margem de erro de 10%^20^ em outras populações, assim como a distribuição obtida (Figura 3). O questionário utilizado demonstrou confiabilidade apropriada^22^. A semelhança da amostra populacional estudada com a população geral foi demonstrada (Tabela 3). Houve pequena diferença (p = 0,05871; Kruskal-Wallis) entre a população estudada e a projeção do censo com relação ao nível de escolaridade, devido possivelmente à seleção de público com acesso à internet inerente à metodologia proposta. O preenchimento do questionário aplicado foi 18% mais lento comparado com o questionário original^13^ (3 minutos e 38 segundos), diferença explicada pelo acréscimo das perguntas sobre fatores e índices de saúde, e a taxa de desistência foi baixa.

A prevalência do lipedema mais aceita na literatura foi estimada por Földi & Földi quando extrapolaram sua experiência clínica para a população geral^23^ e estimaram que até 11% da população feminina poderia ser portadora de lipedema. Em outro estudo, Fife et al.^7^ encontraram 10 a 20%^7^. Segundo Schwahn-Schreiber e Marshall^18^, a prevalência alemã incluindo todos os estágios do lipedema atinge 39% da população. Outros relatos do percentual de pacientes de lipedema entre os pacientes em clínicas de linfedema variam de 8 a 18,8%^16^^,^^24^^,^^25^. Porém, nenhum estudo de prevalência do lipedema mencionado utilizou ferramenta validada. Ao serem diagnosticadas, muitas pacientes lembram-se de outros familiares que apresentam as características da doença, sendo que a incidência familiar varia entre 16 a 45% na literatura^7^^,^^26^.

Considerando a metodologia proposta, o simples aumento (desvio à direita no gráfico, Figura 2) no corte da pontuação do questionário aumenta a probabilidade individual de diagnóstico do lipedema, mas, devido à menor sensibilidade, pode ocasionar maior quantidade de falsos negativos, diminuindo, portanto, a quantidade de doentes identificados na população. Assim, o equilíbrio entre a especificidade e a sensibilidade para estimar prevalência é muito importante. O índice de Youden^27^ é o mais frequentemente sugerido; porém, neste estudo, não se mostrou adequado para determinar o melhor ponto de corte. Por ser uma avaliação populacional, optamos por aumentar a especificidade do teste e a probabilidade de diagnóstico individual, para que a medida da prevalência fosse mais conservadora. Atingimos, assim, a probabilidade diagnóstica de 77,8% (IC95%: 64,2-87,3%) na ferramenta utilizada, com especificidade de 0,88 (IC95%: 0,77-0,95) e sensibilidade de 0,46 (IC95%: 0,31-0,6) (Tabela 2 e Figura 2). Com isso, a prevalência encontrada na população feminina brasileira foi de 12,3% (Tabela 1). A projeção do censo de 2021 estima em torno de 100,5 milhões de mulheres no Brasil, 71.739.082 entre 18 e 69 anos. Aplicando a prevalência estimada, estimamos em torno de 8,8 milhões de mulheres entre 18 e 69 anos com sintomas sugestivos de lipedema.

Dudek et al.^6^, em avaliação com questionário semelhante, avaliaram somente o grupo de mulheres polonesas com suspeita de lipedema, estimando IMC médio de 30,8 kg/cm^2^ (DP = 7,1), com 76,5% classificadas como sobrepeso (26,5%) e obesas (50%). Nosso estudo identificou 67,5% das mulheres com lipedema com IMC maior que 25 kg/cm^2^, sendo o IMC médio de 27kg/cm^2^. O IMC alto dificulta o diagnóstico pela complexidade na diferenciação com a obesidade comum. Pode ser difícil distinguir entre lipedema e outras variações anatômicas da deposição da gordura^28^; afinal, a desproporção da distribuição da gordura típica do lipedema pode ser facilmente confundida com a desproporção ginecoide ou a obesidade em forma de pera^29^, que não apresenta conjunto de sintomatologia do lipedema. O estudo polonês também identificou hipotireoidismo em 31,6%^30^, enquanto outro estudo, na população holandesa, identificou em 11,7%^31^. Nas brasileiras, identificamos essa doença em 19,4%, sem correlação entre voluntárias com ou sem sintomas sugestivos de lipedema.

O linfedema, autorrelatado em 30,6% das holandesas, foi mencionado por apenas 3,2% das brasileiras. A insuficiência venosa, que, nas polonesas, apareceu em 20,4%, nas brasileiras ocorreu em 35,5%, sem correlação entre grupos – apesar disso, 48,4% relataram cirurgia prévia de varizes, o que apresentou correlação positiva. A artrite foi identificada em 20,4% das polonesas. Dores nos joelhos foram autorrelatadas em 58,1% das brasileiras com lipedema. A hipertensão arterial foi identificada em 4%^6^ das portadoras de lipedema polonesas e em 18,4%^31^ das holandesas, porém, nas brasileiras foi mencionado em 41,9%, com correlação positiva. A hipermobilidade articular, característica da síndrome de Ehlers-Danlos^32^, embora previamente publicada como associada ao lipedema^33^^,^^34^, não foi frequente nas populações polonesa^6^ ou holandesa^31^; porém, apesar da baixa frequência, houve correlação positiva em 9,7% das brasileiras.

Em relação aos sintomas associados relatados, foi mencionada a facilidade de ganho de peso em braços e membros inferiores das polonesas em 99%, sensação de peso nas pernas em 96,9%, equimoses frequentes em 90,8% e dificuldade de perder peso em pernas e braços em 86,8%. A dificuldade de perda de peso e volume de membros foi identificada em 51,6% das brasileiras, com correlação positiva. Observamos que a sensação de peso foi citada por 51,6% das brasileiras, com equimoses frequentes em 54,8%, apresentando correlação positiva, assim como a queimação nas pernas. A sensibilidade ao toque foi mencionada no questionário em 35,5% das brasileiras, dor nas pernas em 90,3%, inchaço em 51,6% e sensação de retenção de líquido nas pernas em 64,5%, características que preenchem os critérios diagnósticos atuais^35^.

Na literatura^6^^,^^31^^,^^36^, 42 a 59,2% reportam sintomas depressivos e ansiedade. Encontramos depressão autorrelatada em 38,7% das brasileiras com lipedema e ansiedade em 61,3%, ambas com correlação positiva. A anemia, não previamente relatada em outros estudos semelhantes, foi mencionada por 41,9% das voluntárias e apresentou com correlação positiva com a doença.

Na avaliação polonesa^6^, 34,7% das pacientes relataram qualidade de vida boa ou muito boa, enquanto 20,4% relataram qualidade de vida ruim ou muito ruim. Da mesma forma, encontramos aumento significativo das mulheres brasileiras com sintomas sugestivos de lipedema reportando saúde razoável ou ruim, com correlação positiva, ficando evidente o quanto os sintomas relacionados ao lipedema afetam negativamente na percepção de saúde geral (Figura 4).

Devido ao aumento da divulgação do lipedema na mídia, o diagnóstico do lipedema pode ser tendencioso, sendo necessário considerar os diagnósticos diferenciais^37^, como a lipohipertrofia, obesidade, linfedema, fleboedema, edema de estase e fibromialgia^29^. Apesar disso, o lipedema ainda é muito subdiagnosticado^38^. Os sintomas e as queixas do lipedema podem ser considerados subjetivos, principalmente nas fases mais precoces, e confundem-se com outras doenças muito frequentes no consultório de cirurgia vascular, de modo que a padronização dos critérios de avaliação para aumentar a objetividade do diagnóstico dessa enfermidade é necessária. Por isso, é importante desenvolver e validar instrumentos que possam avaliar o impacto clínico do lipedema e auxiliar no diagnóstico definitivo. A ausência do diagnóstico correto em pacientes com lipedema atrasa o tratamento da doença, permitindo a sua progressão^38^. Os sintomas e histórico associados ao lipedema que demonstraram correlação positiva foram dor nas pernas, “retenção de líquido”, sensibilidade ao toque, inchaço/edema nas pernas, roxos frequentes nas pernas, hipermobilidade das articulações, dor no joelho, sensação de peso nas pernas, queimação nas pernas, dificuldade de perder peso ou volume nas pernas, cirurgia prévia de varizes e lipoaspiração.

Esta pesquisa é a primeira a avaliar a prevalência e as características do lipedema na população brasileira e foi desenhada para obter a prevalência do lipedema no Brasil. Possui algumas limitações, entre elas, a natureza cíclica dos sintomas do lipedema^5^ que pode subestimar a prevalência populacional. Além disso, o estudo foi conduzido de forma on-line, baseado em autoavaliação – o que limita o nível socioeconômico –, e sem supervisão médica ou confirmação diagnóstica.

## CONCLUSÃO

Nesta pesquisa, observamos que a prevalência do lipedema na população de mulheres brasileiras é de 12,3%. Estimamos, de forma conservadora, que 8,8 milhões de mulheres adultas brasileiras entre 18 e 69 anos podem ser portadoras dos sintomas sugestivos do diagnóstico de lipedema. Ansiedade, depressão, hipertensão arterial e anemia parecem estar associadas ao lipedema.
